# Sanitary installations and wastewater plumbing as reservoir for the long-term circulation and transmission of carbapenemase producing *Citrobacter freundii* clones in a hospital setting

**DOI:** 10.1186/s13756-023-01261-9

**Published:** 2023-06-19

**Authors:** Hannelore Hamerlinck, Annelies Aerssens, Jerina Boelens, Andrea Dehaene, Michael McMahon, Anne-Sophie Messiaen, Stien Vandendriessche, Anja Velghe, Isabel Leroux-Roels, Bruno Verhasselt

**Affiliations:** 1 Department of Laboratory Medicine, Ghent University Hospital, Ghent, Belgium; 2 Department of Diagnostic Sciences, Ghent University, Ghent, Belgium; 3 Department of Infection Control, Ghent University Hospital, Ghent, Belgium; 4 Department of Geriatrics, Ghent University Hospital, Ghent, Belgium

**Keywords:** Carbapenemase-producing *Enterobacterales*, CPE, CgMLST, *Citrobacter freundii*, OXA-48, Hospital, Outbreak, Toilet, Wastewater collection system, WGS

## Abstract

**Background:**

Accumulating evidence shows a role of the hospital wastewater system in the spread of multidrug-resistant organisms, such as carbapenemase producing *Enterobacterales* (CPE). Several sequential outbreaks of CPE on the geriatric ward of the Ghent University hospital have led to an outbreak investigation. Focusing on OXA-48 producing *Citrobacter freundii*, the most prevalent species, we aimed to track clonal relatedness using whole genome sequencing (WGS). By exploring transmission routes we wanted to improve understanding and (re)introduce targeted preventive measures.

**Methods:**

Environmental screening (toilet water, sink and shower drains) was performed between 2017 and 2021. A retrospective selection was made of 53 *Citrobacter freundii* screening isolates (30 patients and 23 environmental samples). DNA from frozen bacterial isolates was extracted and prepped for shotgun WGS. Core genome multilocus sequence typing was performed with an in-house developed scheme using 3,004 loci.

**Results:**

The CPE positivity rate of environmental screening samples was 19.0% (73/385). Highest percentages were found in the shower drain samples (38.2%) and the toilet water samples (25.0%). Sink drain samples showed least CPE positivity (3.3%). The WGS data revealed long-term co-existence of three patient sample derived *C. freundii* clusters. The biggest cluster (ST22) connects 12 patients and 8 environmental isolates taken between 2018 and 2021 spread across the ward. In an overlapping period, another cluster (ST170) links eight patients and four toilet water isolates connected to the same room. The third *C. freundii* cluster (ST421) connects two patients hospitalised in the same room but over a period of one and a half year. Additional sampling in 2022 revealed clonal isolates linked to the two largest clusters (ST22, ST170) in the wastewater collection pipes connecting the rooms.

**Conclusions:**

Our findings suggest long-term circulation and transmission of carbapenemase producing *C. freundii* clones in hospital sanitary installations despite surveillance, daily cleaning and intermittent disinfection protocols. We propose a role for the wastewater drainage system in the spread within and between rooms and for the sanitary installations in the indirect transmission via bioaerosol plumes. To tackle this problem, a multidisciplinary approach is necessary including careful design and maintenance of the plumbing system.

**Supplementary Information:**

The online version contains supplementary material available at 10.1186/s13756-023-01261-9.

## Background

Multidrug-resistant organisms (MDROs) pose an increasing threat to global health. Carbapenemase-producing *Enterobacterales* (CPE) have been labelled as a priority 1 (critical) pathogen in the ranking of antibiotic-resistant bacteria by the World Health Organisation [1]. CPE have been reported to be endemic in several areas worldwide [2–4]. A survey in 37 European countries by the ‘European Antimicrobial Resistance Genes Surveillance Network’ in 2018 revealed an endemic situation (stage 5) in 4 countries and inter-regional spread (stage 4) in 11 countries including Belgium [5]. Colonisation with carbapenemase-producing *Enterobacterales* (CPE) is associated with a 16.5% chance of infection, rising significantly in elderly and immunocompromised patients [6–8]. Since CPE are usually multidrug-resistant, infections are often difficult to treat as only few antibiotic options remain available [9]. Clinical outcome in patients is therefore poor and overall morbidity, length of stay and mortality are increased compared to cases infected with non-carbapenemase-producing *Enterobacterales* [6, 10].

Most commonly detected in healthcare associated CPE infections are *Klebsiella pneumoniae, Escherichia coli* and *Enterobacter* spp. [11]. *Citrobacter freundii* is a facultative anaerobic Gram-negative bacillus that is frequently found in soil, water, food and intestines. It is mostly recognised as a harmless contaminant or coloniser. However *Citrobacter* spp. infection can be very severe and difficult to treat, in particular in neonates, older adults and immunocompromised patients [12, 13]. More and more evidence suggests a growing role for *Citrobacter freundii* as a causative species of healthcare facility CPE outbreaks [14–18].

Since CPE colonisation usually occurs in the gastrointestinal tract, contamination of hospital sewage water is not unexpected [19–21]. Hospital sanitary installations (sink, toilet, shower) have been recognised as CPE reservoirs [7, 22]. The watery environment creates ideal circumstances for microbial surface colonisation. Colonised sink traps have shown to disperse bacteria and cause CPE colonisation or infection [23–25]. Also, toilet water has been found to be an environmental reservoir in several healthcare facilities from which various CPE species were transmitted [17, 26–28], including in the burn unit of our own institution [29]. Wastewater collection pipes have been postulated as a possible route of spreading [23, 29]. Little is known however on the extent of the wastewater pipes as a spreading route in a hospital setting. In the present study, CPE *C. freundii* clinical and environmental isolates, taken between 2015 and 2022, were investigated for clonal relatedness using whole genome sequencing (WGS) and subsequent core genome multilocus sequence typing (cgMLST) analysis. By investigating the role of sanitary installations in the patient en-suite bathrooms and of the wastewater drainage system as reservoirs and in transmission, we aim to improve understanding of transmission routes and introduce targeted preventive measures.

## Methods

### Setting

The Ghent University Hospital is a 954-bed teaching hospital in Ghent, Belgium. The geriatric ward studied consists out of 17 patient rooms located on the same side of the corridor: 6 single-bed rooms, 10 double-bed rooms and 1 four-bed room that is no longer in use since the start of the COVID-19 pandemic in 2020. The average number of admissions and hospitalisation days on the ward remained similar over the years, with the exception of a temporary decline in November 2020 when the ward was used to treat SARS-CoV-2 patients. The en-suite bathrooms contain one sink for each patient bed, a common toilet and a common shower all within a distance of 1.5 m (see Additional file 1: Fig. S1). Supply and drainage of (waste)water is grouped per 7 to 9 rooms, combining rooms R01-R09 in one circuit and rooms R10-R16 in another (see Additional file 2: Fig. S2). The last room (R00) is connected to a separate circuit. Grey (sink/shower) and black (toilet) water are transported via different adjacent pipes.

### Design

Because of an unexpected high proportion of *Citrobacter freundii* positive samples at the geriatric ward, a retrospective molecular analysis of bacterial isolates was conducted to gain deeper insights. Ethical approval for this study was obtained by the Ethics Committee of the Ghent University Hospital (ONZ-2022-0085).

### Infection control measures

As a standard of care, at admission to the geriatric ward all patients were screened routinely for CPE with a rectal swab. If a patient became CPE positive during hospitalisation, all other patients were screened weekly as long as the positive patient stayed on the ward. If CPE was only isolated from rectal swab culture it was considered as colonisation, whereas if CPE was isolated from a clinical sample, it was considered as infection. If the patient tested positive for the first time at least 48 h after hospital admission, transmission was considered nosocomial. The pathogen was considered community-acquired if the patient tested positive within 48 h after admission and had not been admitted to the hospital in the last 12 months. The standard room cleaning protocol consists of daily cleaning of the floor and bathroom, using microfiber cloths that are changed per surface. High touch surfaces are disinfected with Clinell® wipes (Gama Healthcare Ltd, London, UK), containing different biocides including a combination of quaternary ammonium compounds and one polymeric biguanide. Patients colonised or infected with CPE are placed on contact precautions in a single-bedroom and are only allowed to leave the room upon medical indication. Following discharge of a CPE positive patient, the room is cleaned thoroughly according to adjusted guidelines, including removal of disposable materials (such as the toilet brush) and decontamination of surfaces and non-disposables using Clinell® wipes. The infection control team gave instruction for daily application of 250 ml of 15° sodium hypochlorite (bleach) in the toilet bowls (containing about 6 L of water) starting from December 2019 until 26 January 2021, corresponding to ≤ 2,000 ppm NaClO. However, after inquiry, the implementation of this measure was executed inconsistently and differed strongly between cleaning staff members. Therefore, effectiveness could not be evaluated.

### Patient samples

Bacterial isolates for whole genome analysis could be retrieved from 30 patients colonised or infected with CPE *Citrobacter freundii* between July 2015 and October 2021. Average age was 85.6 years (± 4.2 SD) and the male/female ratio was 14/16 (47%/53%). Three patients had a CPE infection (2 urinary tract infections, 1 skin and soft tissue infection) and one case was considered community-acquired.

### Environmental samples

Between 2017 and 2021, 385 environmental samples were taken on the geriatric ward. CPE positive rooms were resampled more regularly for follow-up. The focus was on sanitary installations: 76 shower drain samples and 153 sink drain samples were collected using eSwabs (Copan®, city, Italy), and 156 water samples were collected from toilet bowls. Due to the biased nature of the environmental sampling, a considerable number of positive samples derived from the same sites were excluded for further analysis using WGS. Taking into account an even distribution in space and time, 20 *Citrobacter freundii* OXA-48 environmental isolates were selected. Additionally we sampled (using eSwabs) the drainage of the wastewater collection pipes (grey and black water) in 2022 and included three more *C. freundii* isolates for WGS. Wastewater collection pipe sampling points (P01 and P03) are visualised in Additional file 2: Fig. S2.

### Outbreak

In 2018 and 2019, epidemiological investigation by the hospital infection control team identified two separate CPE outbreaks on the geriatric ward of the Ghent University Hospital, with 10 and 5 patients involved respectively. During the first outbreak, insufficient hand hygiene practice and/or inadequate cleaning and disinfection of medical material was presumed as the cause of the outbreak. During the second outbreak, the transmission route was unclear as the CPE positive patients were spread over the whole ward and there were no medical devices used in common which would explain cross-transmission.

### Conventional analysis

All samples were processed and analysed at the in-hospital Lab for Medical Microbiology. Swabs and toilet water samples were processed as described by Heireman et al. [29]. Bacterial identification was performed by ‘Matrix Assisted Laser Desorption Ionization Time of Flight Mass Spectrometry’ (MALDI-TOF MS, Bruker Daltonics, Bremen, Germany) and resistance to carbapenems (meropenem) was detected by the disk diffusion method according to the ‘European Committee on Antimicrobial Susceptibility Testing’ (EUCAST). *Bla*_*KPC*_, *bla*_NDM_, *bla*_GES,_*bla*_OXA−48−*like*_, *bla*_IMP_, and *bla*_VIM_ genes were identified with polymerase chain reaction according to Heireman et al. [29].

### Whole genome analysis

Fifty-three selected isolates were included for shotgun whole genome sequencing. DNA extraction was performed on 1.5 McFarland solutions derived from stored (-80 °C) isolates that were cultured overnight using the QIAamp DNA Blood kit (Qiagen, Hilden, Germany). Extracted DNA was prepped for sequencing either by mechanical shearing to 200 base pairs (bp) fragments with Covaris LE220-plus Focused-Ultrasonicator (Covaris, Inc., Woburn, MA, USA) or via Illumina DNA PCR-Free Prep (Illumina Inc., San Diego, CA, USA). Sequencing was done on a HiSeq 3000 or NovaSeq 6000 instrument (Illumina Inc.) generating 50 bp single end reads. Resulting Fastq-files were filtered and trimmed using fastp [30]. De novo assembly was generated using Spades (isolate settings) [31] and txid was determined with Kraken2 using the MiniKraken database [32]. *In silico* mlst-analysis based on generated assemblies and 522 retrieved NCBI assemblies was done using the PubMLST database [33–35]. Core genome MLST (cgMLST) was performed using chewBBACA [36] with inhouse developed cgMLST-schemes (3,004 loci). Minimum spanning trees (MST) were created using Phyloviz online and GrapeTree [37, 38]. Further analysis (distance matrix, hierarchical clustering) and visualisation was executed with R studio (R version 4.1.2) and iTol [39]. Based on earlier experience inhouse with the selected species and the developed cgMLST-schemes the cut-off for clonal relatedness, and thus cluster formation, was set at 20 alleles. Additionally, isolates with up to 40 allele differences were also considered associated if the isolates were sampled more than a year apart. Isolates with over 40 allele differences were categorised as ‘sporadic’. A cluster was marked as a ‘patient cluster’ if at least two patients were involved.

## Results

**Fig. 1:**
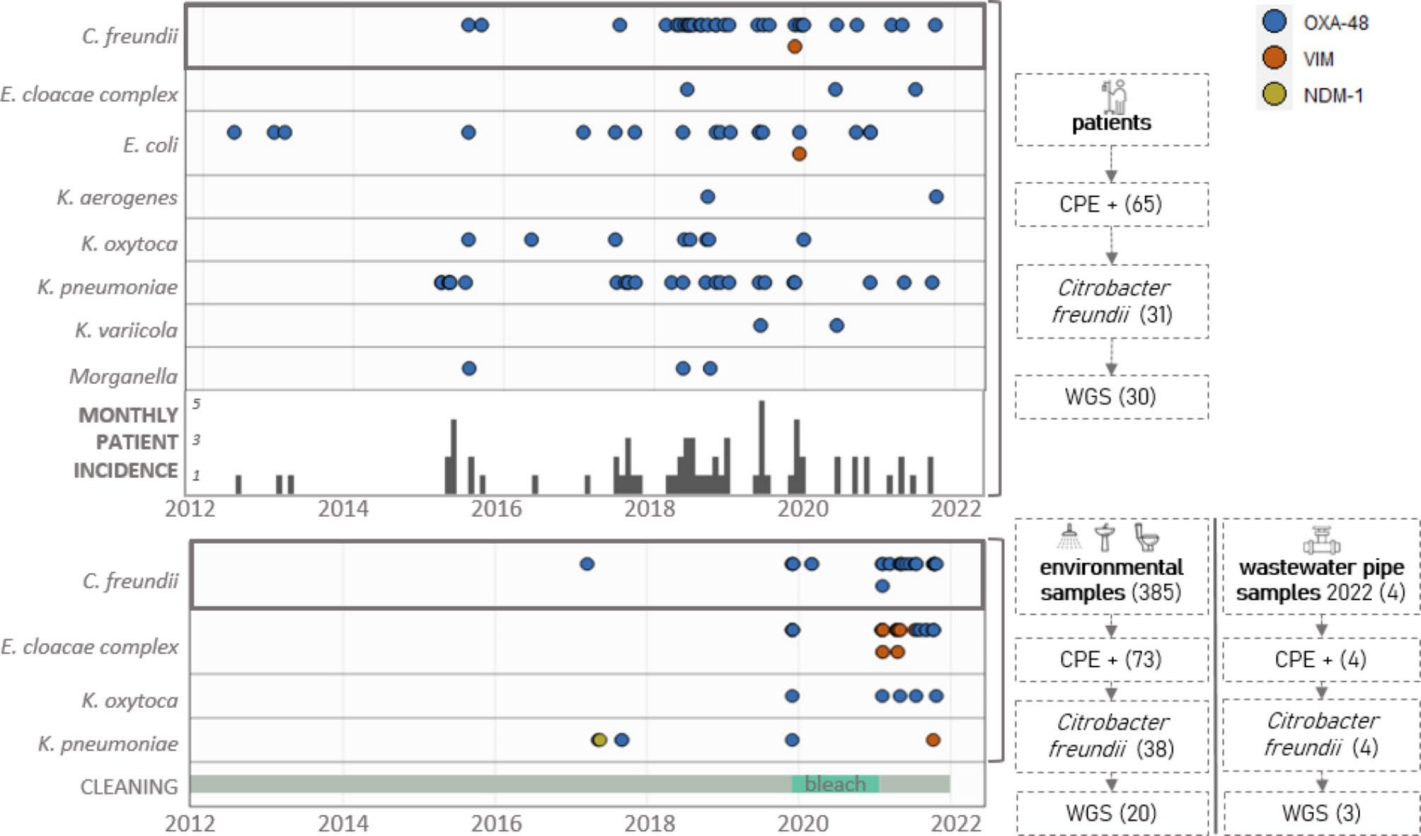
Visualisation of carbapenemase-producing *Enterobacterales* (CPE) per species over time in patient and environmental samples *OXA-48 (blue), VIM (orange) and NDM-1 (yellow) producing bacterial isolates from patients (upper panel) and from environmental samples (lower panel) detected at the geriatric department over a 10-year timespan (2012–2021). Wastewater pipe isolates (2022) are not included in the lower panel. Beside standard cleaning protocols (grey), bleach was used daily in toilets (green) from December 2019 until January 2021. On the right side the flow charts show the number of selected isolates for whole genome sequencing (WGS)*

Over a ten year time period (2012–2021), from 65 unique patients one or more CPE species (93 CPE in total) were isolated on the geriatric ward, of which 14 patients had an infection (13 urinary tract infections, 2 upper respiratory tract infections, 3 skin and soft tissue infections and 1 bloodstream infection). Most of the cases were nosocomial (60/65, 92.3%) and almost half of the patients carried *Citrobacter freundii* (31/65, 47.7%), see Fig. 1. Second and third most prevalent species were *K. pneumonia*e (25/65, 38.4%) and *E. coli* (19/65, 29.2%). The predominant CPE-type was OXA-48 (91/93, 97.8%). In-depth analysis was performed on *Citrobacter freundii* isolates from 30 patients, characteristics can be found in Table 1.

Environmental sampling was performed focusing on toilet water, sink drains and shower drains. A total of 73/385 (19.0%) targeted environmental samples tested positive for one or more carbapenemases, see Fig. 1; Table 2. Highest detection rates were found in the shower drain samples (29/76, 38.2%) followed by toilet water samples (39/156, 25.0%). CPE positivity rates in sink drain samples were 10-fold lower (5/153, 3.3%). *Citrobacter freundii* was the most frequently detected species (38/73, 52.1%), followed by *Enterobacter cloacae* complex (30/73, 41.1%), *Klebsiella pneumoniae* (7/73, 9.6%) and *Klebsiella oxytoca* (5/73, 6.8%). OXA-48 was the predominant carbapenemase (51/73, 69.9%), followed by VIM (27/73, 37%) and NDM-1 (1/73, 1.4%).

**Table 1:** Characterisation of selected environmental and patient *Citrobacter freundii* isolates subjected to whole genome sequencing

Location of detection	Identifier	Sample month	Sample origin	CPE-type	Days on ward until detection	Length of stay on ward	Prior admission(s) on ward (< 12months)	Re- admission(s) on ward (< 12months)	Cluster-type
R00	Patient23	11/2019	Rectal (swab)	VIM	18	40	no	no	spor.
R01	Environment08	12/2019	Shower	OXA-48	-	-	-	-	spor.
	Environment16	5/2021	Toilet	OXA-48	-	-	-	-	spor.
R02	Patient05*	4/2018	Rectal (swab)	OXA-48	1	18	no	yes	C
	Patient08	6/2018	Rectal (swab)	OXA-48	33	112	no	yes	spor.
	Patient26	12/2019	Rectal (swab)	OXA-48	5	8	no	no	C
	Environment09	2/2021	Toilet	OXA-48	-	-	-	-	C
	Environment10	2/2021	Toilet	OXA-48	-	-	-	-	D
R04	Patient03	7/2017	Rectal (swab)	OXA-48	0	1	yes	no	spor.
	Patient28	9/2020	Rectal (swab)	OXA-48	5	8	yes	no	spor.
	Environment11	2/2021	Toilet	OXA-48	-	-	-	-	A
	Patient29	3/2021	Rectal (swab)	OXA-48	9	11	no	no	A
	Patient30	4/2021	Urine	OXA-48	13	16	no	yes	A
	Environment17	5/2021	Toilet	OXA-48	-	-	-	-	A
	Environment21	7/2021	Shower	OXA-48	-	-	-	-	A
R06	Patient01	7/2015	Rectal (swab)	OXA-48	13	31	no	no	B
	Patient02	9/2015	Rectal (swab)	OXA-48	8	14	no	yes	B
	Environment01	3/2017	Toilet	OXA-48	-	-	-	-	B
	Patient09	6/2018	Rectal (swab)	OXA-48	8	9	yes	no	B
	Patient11	7/2018	Rectal (swab)	OXA-48	5	13	no	yes	B
	Patient12	8/2018	Rectal (swab)	OXA-48	8	11	no	yes	B
	Patient13	8/2018	Rectal (swab)	OXA-48	5	11	no	no	B
	Patient15	10/2018	Rectal (swab)	OXA-48	22	25	no	no	D
	Patient16	10/2018	Rectal (swab)	OXA-48	5	26	no	no	spor.
	Patient21	7/2019	Rectal (swab)	OXA-48	8	8	no	no	B
	Patient22	11/2019	Rectal (swab)	OXA-48	1	2	yes	no	B
	Patient24	12/2019	Rectal (swab)	OXA-48	6	8	no	no	spor.
	Environment03	12/2019	Toilet	OXA-48	-	-	-	-	B’
	Environment12	2/2021	Toilet	OXA-48	-	-	-	-	B’
	Environment14	3/2021	Toilet	OXA-48	-	-	-	-	E
	Environment15	3/2021	Toilet	OXA-48	-	-	-	-	B’
	Environment18	5/2021	Toilet	OXA-48	-	-	-	-	spor.
	Environment19	5/2021	Toilet	OXA-48	-	-	-	-	E
R08	Patient10	6/2018	Rectal (swab)	OXA-48	19	29	no	yes	A
	Patient31	10/2021	Abscess	OXA-48	22	48	no	no	spor.
R10	Patient14	9/2018	Rectal (swab)	OXA-48	16	29	no	no	A
	Patient18	1/2019	Rectal (swab)	OXA-48	3	11	no	no	A
	Patient25	12/2019	Rectal (swab)	OXA-48	8	23	yes	no	A
	Environment04	12/2019	Toilet	OXA-48	-	-	-	-	A
	Environment05	12/2019	Toilet	OXA-48	-	-	-	-	spor.
	Environment20	5/2021	Toilet	OXA-48	-	-	-	-	A
R11	Patient20	6/2019	Rectal (swab)	OXA-48	6	8	no	no	A’
	Environment13	2/2021	Shower	OXA-48	-	-	-	-	A
R12	Patient04	3/2018	Urine	OXA-48	13	17	no	yes	A
	Patient07	5/2018	Rectal (swab)	OXA-48	10	11	no	no	A
	Environment06	12/2019	Toilet	OXA-48	-	-	-	-	A
R13	Patient06	5/2018	Rectal (swab)	OXA-48	0	11	yes	no	A
	Environment07	12/2019	Toilet	OXA-48	-	-	-	-	A
	Patient27	6/2020	Rectal (swab)	OXA-48	1	22	no	no	A
R14	Patient17	12/2018	Rectal (swab)	OXA-48	2	39	yes	no	A
P01	Environment22	09/2022	BW pipe	OXA-48	-	-	-	-	A
	Environment24	09/2022	GW pipe	OXA-48	-	-	-	-	B’
P03	Environment23	09/2022	BW pipe	OXA-48	-	-	-	-	spor.

A = ClusterA (ST22), B = ClusterB (ST170), BW = black water, C = ClusterC (ST421), D = ClusterD (ST481), E = ClusterE (ST146), GW = grey water, P = group of wastewater pipes (P01 collects rooms R01 to R09; P03 collects rooms R10 to R16), R = patient room number, spor.=sporadic (> 40 different alleles), ‘ =associated to a cluster (> 20 and < = 40 different alleles), - =not applicable. Isolates are listed according to location of detection, *=community acquired

**Table 2:** Results of CPE screening of 385 environmental samples taken at geriatric ward, 2017–2021

		shower drain	sink drain	toilet water
	<total>	< 76>	< 153>	< 156>
Negative	< 312>	47	148	117
NDM-1	< 1>			
*K. pneumoniae*				1
OXA-48	< 45>			
*C. freundii*		7	1	23
*C. freundii & K. oxytoca*			1	4
*E. cloacae* complex		1	2	1
*K. pneumoniae*				5
VIM	< 21>			
*E. cloacae* complex		16		4
*K. pneumoniae*		1		
VIM & OXA-48	< 6>			
*E. cloacae* complex		3	1	
*E. cloacae* complex *& C. freundii*		1		1

**Fig. 2:**
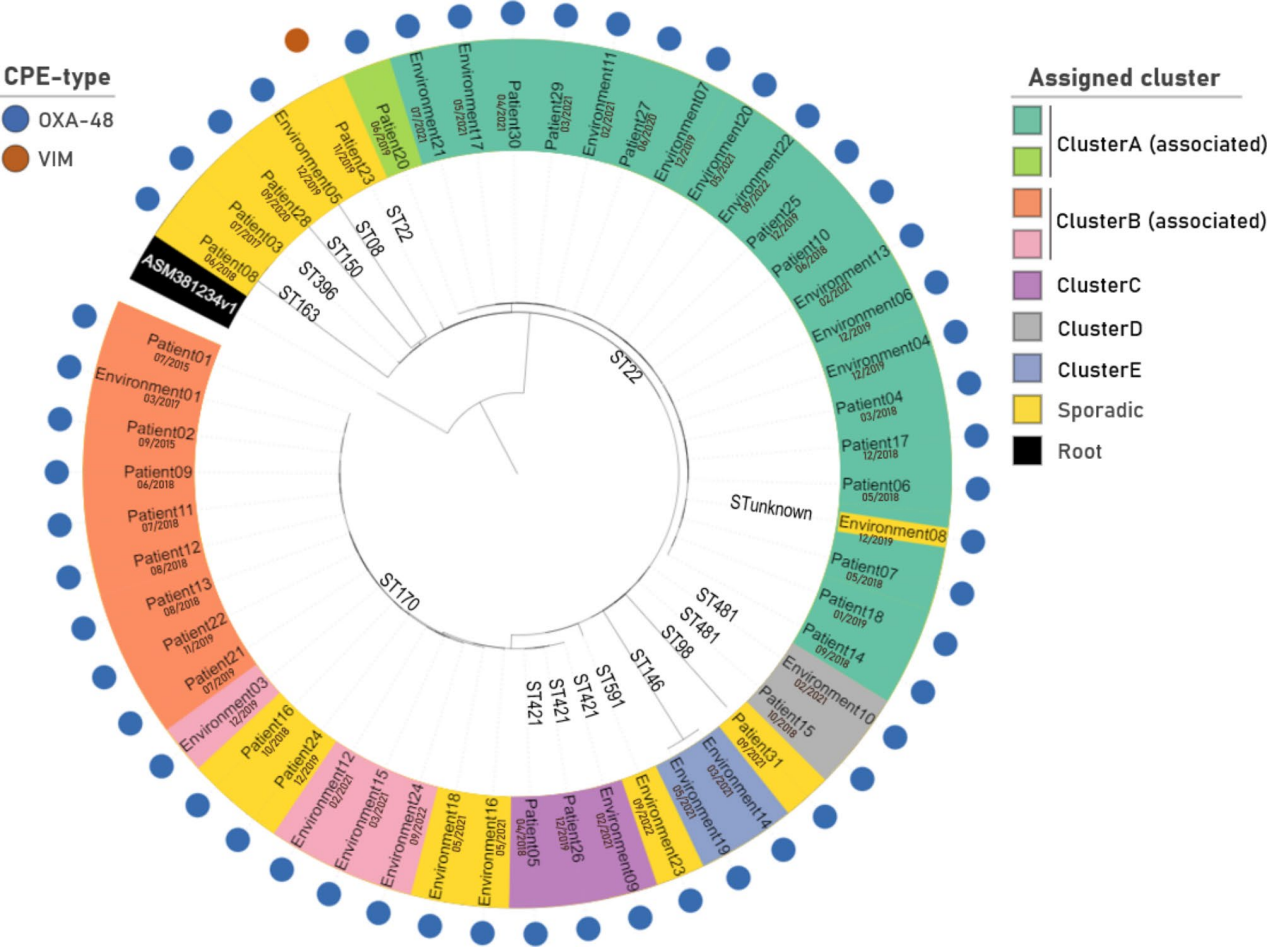
Cluster analysis of 53 *Citrobacter freundii* isolates *Cladogram visualising cgMLST hierarchical clustering results, sampling dates and sequence types (ST) from 53 selected Citrobacter freundii isolates and NCBI assembly ASM381234v1 (root). Isolates that have been assigned to a cluster have < = 20 allele differences and isolates in an associated cluster have < = 40 allele differences based on minimum spanning tree analysis. All others (> 40 allele differences) are categorised as ‘sporadic’. Hierarchical clustering analysis fails to confirm cluster assignment by minimum spanning tree analysis of isolates ‘Patient16’, ‘Patient24’ and ‘Environment08’.*

**Fig. 3:**
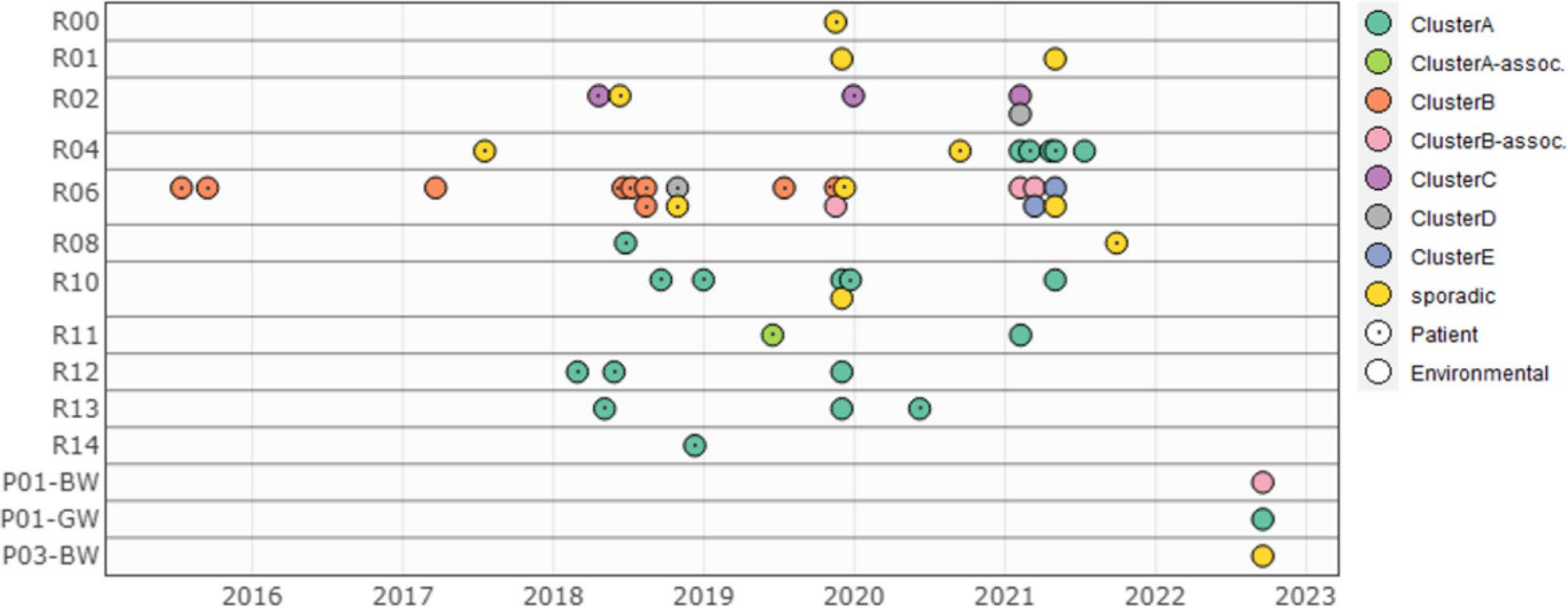
Visualisation of 53 *Citrobacter freundii* isolates in time according to collection site *BW = black water, GW = grey water, P = group of wastewater pipes (P01 collects rooms R01 to R09; P03 collects rooms R10 to R16), R = patient room number. The isolates were coloured by assigned cluster and visualised in time (x-axis = time point of collection) and space (y-axis = site of collection). Patient isolates are indicated with an inner dot*

To investigate clonal relatedness between patient and environmental isolates we performed WGS on 53 *Citrobacter freundii* isolates (30 patients, 23 environmental samples), characteristics see Table 1. On average 8.8 million reads were retained after filtering and trimming steps, resulting in an average coverage of 88x. Mean N50 of the assemblies was 103,840 bp and average number of contigs was 160. Clustering based on the minimum spanning tree created after cgMLST analysis revealed 12 cases in which no clonal relation could be found (“sporadic”), see Fig. 2. The remaining 41 isolates were clustered into 5 different clusters involving both patient and environmental isolates; 2 large clusters (ClusterA: ST22, ClusterB: ST170) and 3 smaller clusters (ClusterC: ST421, ClusterD: ST481, ClusterE: ST146). Figure 3 shows an overview of all sequenced isolates in time per sampling location. Three out of 5 detected clusters were labelled as patient clusters involving at least 2 different patients (ClusterA, ClusterB and ClusterC). The presence of environmental samples in all five clusters suggests that sanitary facilities may have played a role in the circulation of these clones.

**Fig. 4:**
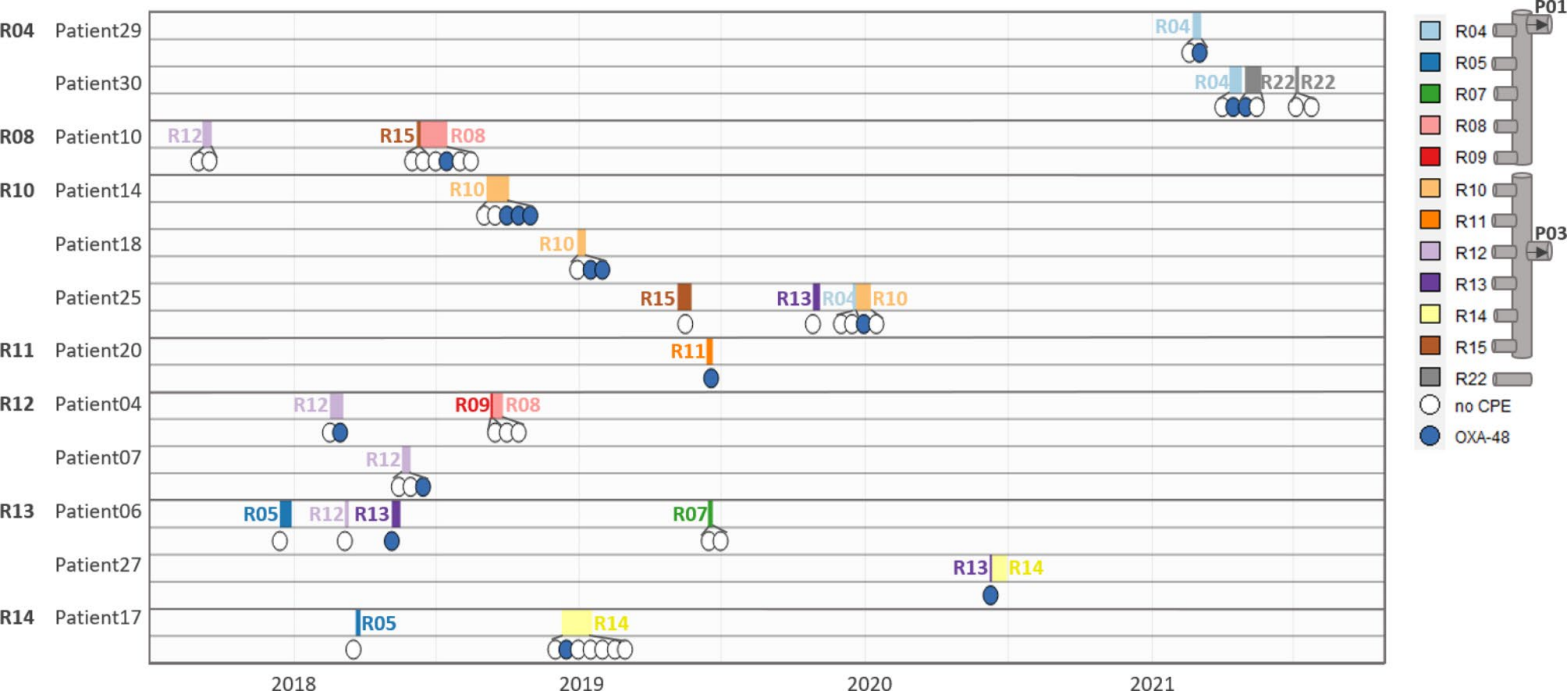
Room occupation and CPE status of patients belonging to the ST22-cluster (ClusterA) over time *P = group of wastewater pipes (P01 collects rooms R01 to R09; P03 collects rooms R10 to R16), R = patient room number. Two lanes per patient, the first lane (blocks) visualises the room(s) occupied, the second lane (circles) summarises CPE status over time. Patients are grouped according to the room number of first CPE detection. Wastewater drainage system is schematically represented in the legend (identical for both grey water as black water)*

ClusterA (ST22) contains 21 isolates of which 20 show clonal relatedness ( < = 20 allele differences) and 1 is associated ( < = 40 allele differences), see Figs. 2 and 3 and Additional file 3: Fig. S3.A. The cluster involves 12 patient cases, hospitalised between 2018 and 2021. All patient cases were categorised as nosocomial. During the years studied the ClusterA-clone seems to gradually move up the hallway, starting from the rooms in the second half of the hallway to the rooms at the beginning. This trend cannot be explained by patient movements or the sharing of (medical) equipment, see Fig. 4. In silico MLST-analysis of 522 NCBI available *Citrobacter freundii* genomes revealed ST22 as the most common strain type [35], therefore (re)introduction via external sources is possible. However because the patients were CPE negative at admission and because of the high resolution of cgMLST typing applied, several introductions via external sources (e.g. residential care centres) are considered unlikely. Most patient cases were found to be several months apart from one another; therefore, we considered an internal environmental reservoir. Six toilet water samples and two shower drain swabs were shown to match with the same *Citrobacter freundii* clone, indicating involvement of the sanitary installations in the room. In room R04 the presence of the ClusterA-clone could be confirmed in toilet water weeks before the first ClusterA-patient was diagnosed in the room (2021). Additionally the same clone was detected in 2022 in an environmental swab taken from the grey wastewater collection pipe draining the first part of the ward (rooms R01 to R09). Three out of twelve patients connected to ClusterA were staying in one of those rooms at primary detection. It must be noted that room transfers are common and that 5 patients (Patient04, Patient06, Patient10, Patient17, Patient25) resided in rooms on both sides of the ward (rooms R01-R09 / rooms R10-R16), see Fig. 4.

**Fig. 5:**
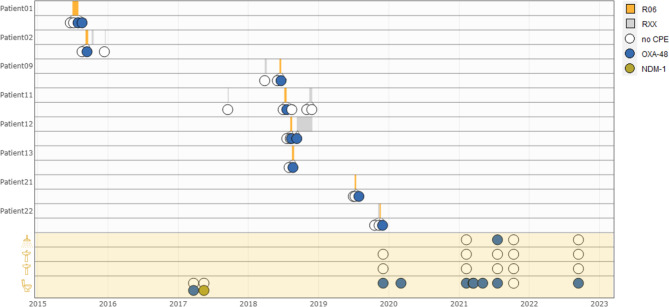
Room occupation and CPE status of patients belonging to the ST170-cluster (ClusterB) over time *Upper panel (white) visualises patient implicated in the ST170-cluster. There are two lanes per patient; the first lane (blocks) visualises room occupancy (RXX = room number other than R06) and the second lane (circles) summarises CPE status over time. Lower panel (orange) visualises CPE status of environmental samples taken at R06*

ClusterB (ST170) contains at least 13 isolates of which 9 were closely related ( < = 20 allele differences) and 4 more distantly ( < = 40 allele differences) (Fig. 2 and Additional file 3: Fig. S3.B). All but one of the samples were taken in the same room (R06) (Fig. 3). The first isolate originates from a patient in 2015 and the last one is derived from a black wastewater collection pipe sample (connected to rooms R01-R09) taken in 2022. In total 8 patients, categorised as nosocomial, were connected to this cluster. A time span of 3 years separates the second and third patient (2015 to 2018). Recurrence of the clone in 2018 could not be explained by reintroduction by a previous resident since none of the affected patients returned to the room afterwards, see Fig. 5. In 2017, the same ClusterB-clone was detected in the toilet water of room R06. An associated clone was found again in the toilet water in 2019 and in 2021, indicating the presence of a toilet reservoir. A similar situation can be seen in ClusterC (ST421), this cluster contains 2 patient and 1 toilet isolate, all taken in the same room (R02). The samples were collected over a year apart from each other (2018, 2019 and 2021).

## Discussion

CPE transmission is an increasing problem in the healthcare facility setting. In the geriatric ward of the Ghent University Hospital, we detected 65 CPE patient cases between 2012 and 2021, mainly OXA-48 producing *Citrobacter freundii*. A retrospective study using WGS data revealed long-term co-existence of three different OXA-48 producing *Citrobacter freundii* clusters in close proximity (ST22, ST170 and ST421) despite infection control measures (contact precautions) and appropriate cleaning protocols.

The clusters detected involve both patient as well as environmental isolates (toilet water, shower drains, sink drains and wastewater collection pipes). Water reservoirs play an important role in the transmission of CPE in healthcare facilities. This has been illustrated in a meta-analysis by Gordon et al. that revealed drains/drainage systems and sinks surfaces as the most frequently colonised [7]. In our study, shower drain and toilet water samples were significantly more contaminated than sink drain samples. Using WGS-analysis we were able to perform molecular typing for 53 isolates (30 patients and 23 environmental samples) with high sensitivity. The analysis revealed two clusters that were each associated with one specific room (ClusterB: ST170 and ClusterC: ST421). The environmental sampling revealed persistent presence of the same clone in the toilet water. It must be noted that colonisation of the toilet can be merely due to patient shedding in faeces and urine; the toilet may therefore serve as a receiver more than a spreader [40]. However, the same clone was detectable in the toilet for long periods, often longer than a year, despite the absence of CPE positive patients in the room. These findings are indicative for a persistent toilet reservoir. Moreover, we suspect the toilets to cause transmission via the formation of bioaerosol plumes as has been described in other CPE outbreaks as well [17, 26, 29]. By toilet flushing and subsequent bioaerosol plume formation (pathogenic) bacteria can spread in the area (> 0.5 m) and stay in the air for extended periods of time (> 15 min) leading to transmission via skin contact, surface contamination or inhalation [41, 42]. Additionally transmission can occur via the spreading of droplets during toilet flushing and urination [13, 43]. A study by Klein et al. suggests a short contact time (e.g. 1 toilet visit) may be sufficient for MDRO transmission since they found 4 unrelated patient isolates to be connected to toilet isolates in an outpatient clinic [28]. In a recent manuscript published during revision of our paper, Neidhöfer et al. confirm that sanitary facilities play an important role in maintaining a reservoir for MDRO over many years, particularly in wards with high antibiotic exposure [22].

The largest detected CPE cluster (ClusterA = ST22) was spread over the complete ward but mainly localised in the second half (R10-R16). A large part of the transmissions could not be explained by patient-to-patient transmission because of long intervals between stays, often several months. Earlier research in our hospital revealed the wastewater pipes as a possible route for CPE cross-transmission between rooms via the retrograde migration of biofilms [29]. We suggest a similar mechanism for the spread of the OXA-48 producing *Citrobacter freundii* ST22-clone. We presume inter-room transmission of ClusterA clones to have occurred via the retrograde formation of biofilm in the wastewater pipes in at least 3 situations (Fig. 2); the transmission of the clone to room R10 where the first occurrence in a patient (Patient14) was detected in September 2018; the transmission of the clone to Patient20 residing in room R11 where no other ClusterA patient ever resided and at a time where no other ClusterA patients had been detected at the ward for 5 months (June 2019) and finally a ClusterA clone was detected in the toilet water of room R04 (2/2021) before the first ClusterA patient in that room had been diagnosed (3/2021). This hypothesis was confirmed again by sampling of the wastewater collection pipe across the hallway in which the same ST22 clone was found in 2022 over one year after the last patient was detected. Distance from the main collecting pipe to the respective toilets and showers across the hallway is about 4 m. Research with GFP-expressing *Escherichia coli* strains has shown that spread via biofilm from collecting pipes to sink drains in a hospital setting can occur at a speed of about 2.5 cm per day [23]. Theoretically, a distance of 4 m could be covered in less than 6 months, which seems realistic given the timeframe of events in our study. To our knowledge, a CPE biofilm spread of several meters through drainpipes has not yet been described. The geriatric ward wastewater system is divided in two circuits splitting rooms R01 to R09 from rooms R10 to R16. The outbreak started in the second half of the ward and possibly the transfer between the two wastewater circuits was made via a patient re-localisation (Patient10 moving from room R15 to room R08). The spread of ClusterA might even be wider and more persistent than expected since additional analysis revealed the same clone in a different ward on another floor of the same building. Also, recent screening in 2022 revealed a new patient isolate connected to the geriatric ward belonging to the cluster. These data have not been included in this paper.

Three isolates were labelled as “sporadic” by MST-analysis but were grouped with ClusterA (Environment08) and ClusterB (Patient16 and Patient24) using hierarchical analysis. The latter two isolates, assigned to ST170, were collected from patients hospitalised in room R06 which was the main location for ClusterB isolates. Moreover, both patients were diagnosed during periods of CPE revival in the room. Since ST170 is not a prevalent sequence type for *Citrobacter freundii* the assignment of these isolates as “sporadic” can be disputed. Possibly the three isolates were derived from the respective clusters but experienced increased mutagenesis. Mutation rates may rise significantly under stress conditions such as the use of biocides (e.g. bleach) and antibiotics, which were used intensively at the department [44–47]. Given the specific technical characteristics of our data, including the use of short 50 bp single end reads, the use of de novo assembly and the use of a scheme with 3,004 loci, we have set a clustering cut-off at 20 alleles. This cut-off is higher than those used in other CPE cgMLST outbreak studies, such as *Klebsiella pneumoniae* and *Escherichia coli*, which typically range between 10 and 15 alleles [48–50]. We were unable to find any specific cut-offs for *Citrobacer freundii* cgMLSt analysis in literature, likely due to the limited information available on the species mutation rate and the lack of freely available cgMLST schemes. It’s important to note that the optimal cut-off value for clustering can vary depending on several factors, including the species, mutation rate, technical quality, cgMLST scheme utilized, and timing and continuity of sampling. To account for long time periods between samples, we have introduced a second cut-off of 40 alleles for samples taken more than 1 year apart. This allows compensation for the mutations generated over the years and therefore reduces the likelihood of false negatives. To improve cgMLST interpretation in general we suggest to use additional tools such as the generation of a distance matrix and hierarchical clustering. Data should ideally be interpreted within the context of a larger and technically similar dataset. Finally we suggest to standardise bioinformatic workflows, including the use of cgMLST schemes and species specific cut-offs, to improve comparison of data between healthcare facilities [51].

Clonal dissemination is not the only driver in the spread of carbapenemase genes. The highly conserved *bla*_OXA−48_ gene, typically located on a IncL/M-type plasmid, can be transferred horizontally between *Enterobacterales* species [52] and the hospital sewage system is an ideal hotspot for transfer of resistance genes [53]. It is therefore important to keep in mind that this study, which focuses on OXA-48 producing *C. freundii*, may be part of a more complex tangle of transmission events involving several species and clones. The pattern of most commonly detected species in patients does not seem to mirror that of environmental samples. *Enterobacter cloacae complex* species for example were found in 41.1% of CPE positive environmental samples but rarely in patients (4.6%). Parallel WGS analysis (data not shown) also revealed a widespread environmental contamination with a VIM-producing *Enterobacter hormaechei* clone on the geriatric ward without any patient cases occurring. Several pathogenic bacteria are known to colonise the (hospital) wastewater system [54] but this does not necessarily seem to reflect in patient colonisation or infection.

The year 2020 was clearly marked by fewer patient cases of CPE. The number of hospitalisations at the geriatric department did not drop significantly during the SARS-CoV-19 pandemic but the increased compliance to hand hygiene and cleaning protocols may have had some effect on CPE transmissions. Alternatively, the drop in cases could be explained by the daily disinfection of toilets with a dilute solution of sodium hypochlorite (bleach, ≤ 2000 ppm NaClO) which started in December 2019 until January 2021. Regular cleaning with bleach or acetic acid has shown to have a temporary effect on the eradication of resistant microorganisms but complete replacement of contaminated reservoirs is usually considered the most effective [7, 24, 55, 56]. However, replacement of the sanitary installations does not guarantee success because of the high risk of recolonisation via patient shedding or via retrograde migration of biofilms in the sewage system [28]. As an experiment, in one room (room R04) a new, rimfree toilet was installed in September 2021, nonetheless the toilet was recolonised within 2 weeks. More drastically, the complete removal of sinks and implementation of water-free patient care have shown to be beneficial in an intensive care unit (ICU) [24]. However, this approach has its drawbacks; handwashing is considered superior over alcohol-based hand rub for removal of non-enveloped viruses and bacterial spores (e.g. *Clostridioides difficile)* [57]. Moreover ensuring patient comfort and care, these measures are not easily implemented in a regular hospitalisation ward. A randomised controlled trial in 32 sink and shower drains concluded that a combination of chemical, mechanical and heat cleaning up to the P-trap level results in the best short-term decontamination [56]. But mechanical and heat cleaning are difficult to implement in shower and toilet P-traps.

Since complete elimination of CPE reservoirs has proven to be difficult in our hospital we focussed on reduction of exposure. Washing without water, by the use of cleansing wipes, is implemented at the ward as a standard of care. It must be noted that the showers were already rarely used by the geriatric population. Additionally, during the first months of the pandemic (May 2020) printed stickers “Please close the toilet lid when flushing” were hung on all toilets to diminish the dispersion of secreted microorganisms to surrounding surfaces [58, 59], however it is unclear how well this instruction was followed. Hospital bathrooms are often small with the sinks, toilet and shower in close proximity. This can result in cross-contamination of sanitary installations via bioaerosol plumes. Therefore, toilets are best installed in a separate space, or alternatively the installation of splash screens can provide a physical barrier. A new technique to diminish bioaerosol and droplet dispersion from the water in the toilet bowl is the creation of a foam layer as described by Arena et al. [13]. In order to tackle the problem of biofilm spreading, hospital plumbing design and maintenance is critical. Biofilm-formation is facilitated by stagnating water, pipe corrosion, warm room temperatures and available nutrients [7, 23, 55, 60, 61]. The plumbing system and the use of sanitary installations should therefore be evaluated and improved where possible, this may be facilitated by guidelines such as those distributed by the Centers for Disease Control and Prevention (CDC) [62]. Finally healthcare worker behavior is also considered as a crucial factor, this includes compliance with hand hygiene guidelines but also the correct usage of the sanitary installations. For example analysis of 2748 sink usage videos categorized 56 activities in which nutrients were disposed in the sink, which can promote bacterial biofilm growth [63].

A lot of research is being done on new decontamination techniques. Ultraviolet (UV) light has shown to have a reducing effect on the bioburden [64, 65]. Since UV-C light is already being used for the purification of drinking water it could also be considered for toilet water and associated water droplets [66]. It should however be noted that UVC light as a sole disinfectant treatment can prevent but not eliminate mature biofilms and mechanical cleaning is still necessary. A newer method to degrade biofilms and ‘weaken’ pathogens in general is the use of quorum sensing inhibitors (quorum quenching) [67, 68]. To prevent quick recontamination after cleaning, continuous room decontamination techniques could be practiced. These methods are under investigation and may include far-UV-C light, visible light disinfection, dry hydrogen peroxide, self-disinfection surfaces, continuously active disinfectants and multi-jet cold air plasma [69].

This study has several limitations. First, it does not include the sampling of dry surfaces and supplies (e.g. bed, sink, toilet bowl…) [27]. Contamination can occur through direct patient contact or via (toilet) bioaerosols and droplets. It should be mentioned that *Enterobacterales* have shown to survive on dry surfaces up to several weeks [41] [70]. Second, it is important to note that the positivity rates obtained from environmental screening (sink drain, shower drain, toilet water) are an overrepresentation because of targeted sampling in rooms with previous positive CPE measurements. Furthermore, we focus on the wastewater system and did not pursue anterograde contamination via incoming water. Third, patient data in this study only include laboratory results and room occupation. Alternative data, such as inter-ward transfers, the use of catheters, washing routines…, were not collected since an environmental cause was strongly suspected. It remains possible that certain bacterial strains were re-introduced via the regular patient-transfers between partner care-facilities or the readmission of previously discharged patients [71]. However we consider this chance relatively small given the strictly applied admission screening and the fact that 92.3% of the cases were categorised as nosocomial transmission (> 48 h after hospital admission).

Other study pitfalls include the sequencing of single end 50 bp fragments, these short and unpaired fragments complicate the generation of an accurate assembly especially when dealing with repeating elements. However through deep sequencing we generated coverage levels that exceeded the 40x threshold that has been suggested appropriate for cgMLST analysis [51]. To improve assembly quality (e.g. genomic structure) we should perform long read sequencing and generate hybrid assemblies [72]. Using a sequencing technique, analysis is always prone to genuine minor variants, sequencing and assembly errors. These may result in a higher number of allele differences and by extension a different cluster categorisation, rather than false-positive co-clustering.

## Conclusions

This retrospective study strongly suggests the involvement of the sanitary installations and wastewater pipes in the spreading and transmission of CPE in a hospital setting. Using cgMLST, clonality was shown in five clusters of OXA-48 producing *Citrobacter freundii*. Three clusters included both patient as well as toilet water and shower drain isolates. Indirect transmission is likely to occur via the generation of bioaerosol plumes and droplets while flushing, urinating or showering. In over one third of rooms in the geriatric ward studied, the toilet water or shower drain were proven to be contaminated with the same *Citrobacter freundii* clone. Despite contact isolation measures and cleaning and disinfection procedures involving bleach, spreading seems to have occurred gradually over several years. Moreover isolates of the same clone were detected in the wastewater collection pipe. Therefore we suggest that inter-room spread occurred via the wastewater drainage system through retrograde formation of biofilms. A multidisciplinary approach is needed to tackle this complex problem, including the improvement of use, cleaning, design and maintenance of plumbing and sanitary installations within healthcare facilities.

## Electronic supplementary material

Below is the link to the electronic supplementary material.

**Additional file 1**: **Fig. 1.** Floor plan of sanitary installations in one- (A) and two- (B) patient bathroom. (PNG 128 kb)

**Additional file 2**: **Fig. S2.** Schematic representation of wastewater circuits (black: toilet water and grey: shower/sink water) and sampling points (P01, P03). (PNG 149 kb)

**Additional file 3**: **Fig. S3.** Minimum spanning tree of cgMLST results of ST22 (A) and ST170 (B) *Citrobacter freundii* isolates. (PNG 83 kb)

## Data Availability

The datasets supporting the conclusions of this article are included within the article (and its additional files), assemblies are available in the GenBank repository, PRJNA881267 (https://dataview.ncbi.nlm.nih.gov/object/PRJNA881267).

## Abbreviations

CDC: Centers for Disease Control and Prevention
cgMLST: Core genome multilocus sequence typing
CPE: Carbapenemase-producing *Enterobacterales*
EUCAST: European Committee on Antimicrobial Susceptibility Testing
MALDI-TOF MS: Matrix Assisted Laser Desorption Ionization Time of Flight Mass Spectrometry
MDRO: Multidrug-resistant organism
MLST: Multilocus sequence typing
MST: Minimum spanning tree
R: Room
SD: Standard Deviation
ST: Sequence type
WGS: Whole genome sequencing
