# Supplementary figures and images for: Sanitary installations and wastewater plumbing as reservoir for the long-term circulation and transmission of carbapenemase producing Citrobacter freundii clones in a hospital setting
Source: Antimicrob Resist Infect Control. 2023 Jun 19;12:58. doi: 10.1186/s13756-023-01261-9 (PMC10280848; doi:10.1186/s13756-023-01261-9)

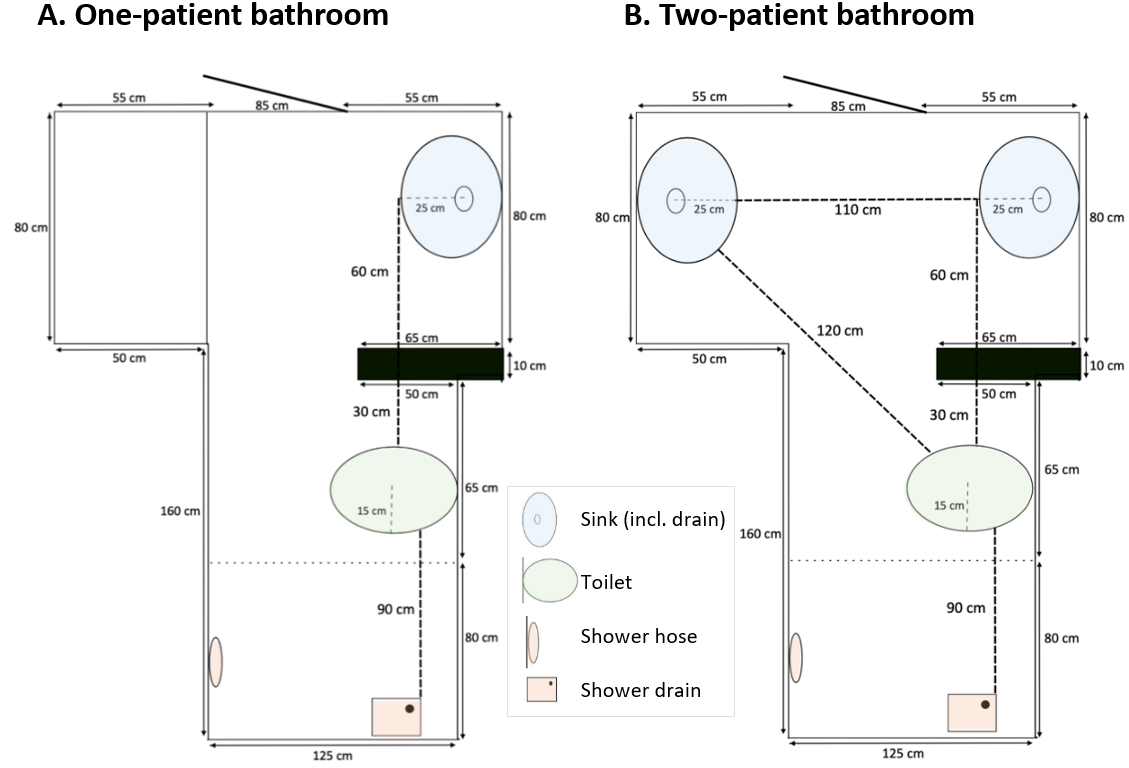

Supplement: Supplementary file 1 — Additional file 1: Fig. 1. Floor plan of sanitary installations in one- (A) and two- (B) patient bathroom. (PNG 128 kb) [file 13756_2023_1261_MOESM1_ESM.png]

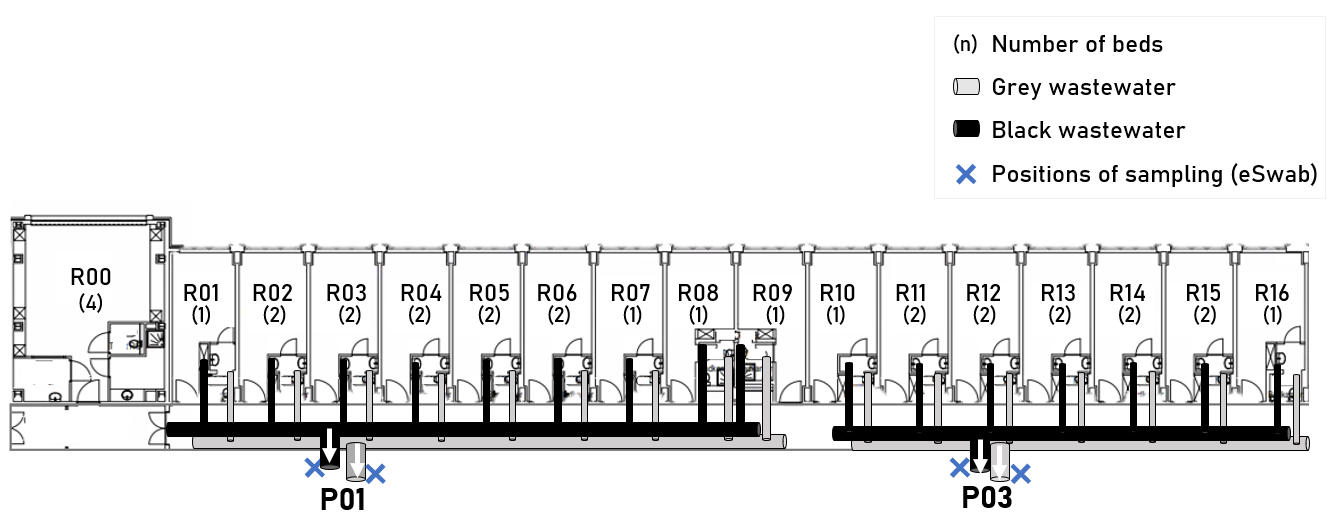

Supplement: Supplementary file 2 — Additional file 2: Fig. S2. Schematic representation of wastewater circuits (black: toilet water and grey: shower/sink water) and sampling points (P01, P03). (PNG 149 kb) [file 13756_2023_1261_MOESM2_ESM.png]

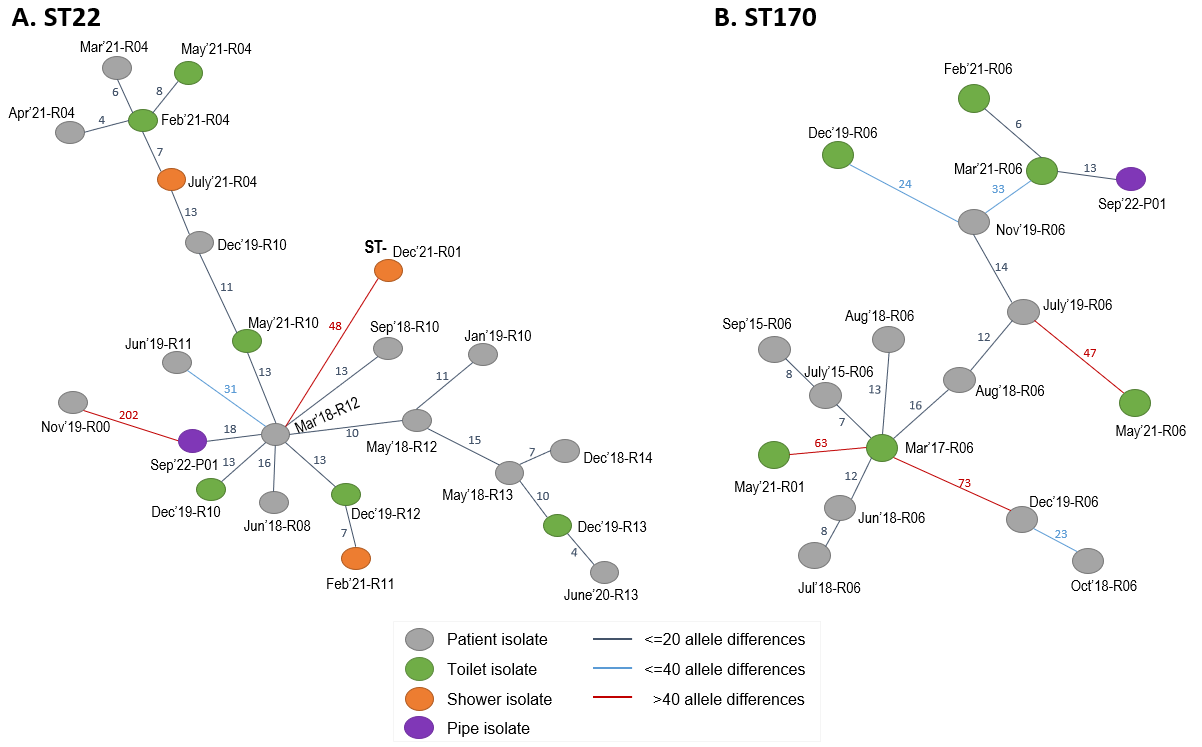

Supplement: Supplementary file 3 — Additional file 3: Fig. S3. Minimum spanning tree of cgMLST results of ST22 (A) and ST170 (B) Citrobacter freundii isolates. (PNG 83 kb) [file 13756_2023_1261_MOESM3_ESM.png]
